# The association between childhood trauma and emotion recognition is reduced or eliminated when controlling for alexithymia and psychopathy traits

**DOI:** 10.1038/s41598-024-53421-5

**Published:** 2024-02-10

**Authors:** Holly Cooper, Ben J. Jennings, Veena Kumari, Aiyana K. Willard, Rachel J. Bennetts

**Affiliations:** Division of Psychology, College of Health, Medicine, and Life Sciences, Brunel University London, Uxbridge, UB8 3PH UK

**Keywords:** Human behaviour, Perception

## Abstract

Emotion recognition shows large inter-individual variability, and is substantially affected by childhood trauma as well as modality, emotion portrayed, and intensity. While research suggests childhood trauma influences emotion recognition, it is unclear whether this effect is consistent when controlling for interrelated individual differences. Further, the universality of the effects has not been explored, most studies have not examined differing modalities or intensities. This study examined childhood trauma’s association with accuracy, when controlling for alexithymia and psychopathy traits, and if this varied across modality, emotion portrayed, and intensity. An adult sample (N = 122) completed childhood trauma, alexithymia, and psychopathy questionnaires and three emotion tasks: faces, voices, audio-visual. When investigating childhood trauma alone, there was a significant association with poorer accuracy when exploring modality, emotion portrayed, and intensity. When controlling for alexithymia and psychopathy, childhood trauma remained significant when exploring emotion portrayed, however, it was no longer significant when exploring modality and intensity. In fact, alexithymia was significant when exploring intensity. The effect sizes overall were small. Our findings suggest the importance of controlling for interrelated individual differences. Future research should explore more sensitive measures of emotion recognition, such as intensity ratings and sensitivity to intensity, to see if these follow accuracy findings.

## Introduction

Emotion recognition is a crucial ability that we employ in our daily interactions and relationships. As social beings, our socialisation and relationships are fundamental to our health and well-being^1^, and expressions of emotion are a core function of social interactions and facilitate appropriate responding in social situations^2^. There are social advantages of more accurate emotion recognition; for example, individuals with higher accuracy are considered more likeable^3^. Further, our perception and classification of emotional expressions influences our behaviour (the emotions as social information model^4^) by providing cues about our social context. For example, recognising a negative expression (sadness or anger) could potentially alerts us that we may be behaving insensitively, leading us to modify our future behaviour. Hence, accurate emotion recognition is a vital ability, as misclassifications may lead to socially inappropriate responses.

Research on individual differences in emotion recognition aims to investigate variations in personal factors (e.g. disorders or traits) that are associated with better or poorer emotion recognition accuracy. Experiences of childhood trauma (e.g. neglect or abuse) are one factor that has frequently been associated with poorer emotion recognition^5^; however, the breadth of those effects (i.e. whether they apply across all emotions and modalities), and their relationship with other individual differences is under-explored. As such, the current study explores the effect of childhood trauma on emotion recognition accuracy, whilst controlling for alexithymia and psychopathy. We explored these relationships across modalities, via the use of video stimuli presented with different intensities.

### Childhood trauma and emotion recognition

Childhood trauma can be defined as exposure to actual or threatening behaviour, serious injury, or sexual violence, and encompasses both neglect and abuse^6^. Childhood trauma has been associated with heightened emotional reactivity, low emotional awareness, and difficulties in regulating emotions^7^. Childhood trauma is also associated with differences in recognising others’ emotions; however, these are not uniform. The effects of childhood trauma on emotion classification accuracy are dependent on the emotional valence of the face; accuracy is higher for negative emotions and poorer for positive or neutral emotions, which are often misclassified as negative^5,8^.

The variable effects of childhood trauma on emotion recognition have been explored in both child and adult samples. Pollak et al.^9^ found that neglected and physically abused children used more liberal criteria when recognising sad and angry facial expressions, respectively, and showed less distinction between angry, sad, and fearful emotional expressions. Bérubé et al.^5^ conducted a systematic review of 24 studies exploring childhood trauma and emotion recognition accuracy in an adult sample. They reported that childhood trauma was associated with variation in performance for the emotions of happiness, anger, and fear. Specifically, happy expressions were less accurately recognised, but anger and fear were recognised faster and at a lower intensity for adults with experience of childhood trauma compared to adults with no experience. However, a meta-analysis^10^ exploring childhood trauma and emotion processing of facial expressions of emotion reported different findings. Saarinen et al.^10^ explored children’s and adults’ neurophysiological and behavioural responses, including accuracy and reaction time (RT). They included 29 behavioural studies and found that childhood trauma experience was associated with faster RTs but “normal” accuracy to sad and angry facial expressions. Childhood trauma was also associated with poorer accuracy for fearful and happy facial expressions, but only if individuals had recent (within the last 2 years) experience of childhood trauma. Therefore, the findings suggest that only the child samples included in the analysis showed variation in accuracy across emotion portrayed.

The increase in sensitivity towards recognition of negative emotion cues found in previous research might be explained by the social information processing mechanism^8^. This states that trauma leads to processing biases for threatening cues/emotions, so recognition is possible with reduced perceptual information. It also suggests that neutral or positive emotions may be misclassified as negative emotional expressions. In support of this mechanism^8^, there is evidence of children with trauma experience being able to recognise negative expressions (anger and fear) with less perceptual information compared to children without trauma experience^9^. This suggests a heightened sensitivity specifically for negative, or threatening, expressions. There is support in adult samples for this trend, suggesting this effect has long-lasting effects into adulthood^50^. Beyond behavioural measures, there is also evidence of increased amygdala activation in individuals with childhood trauma experience when viewing negative expressions compared to individuals without childhood trauma^11^.

The majority of research has focused on facial expressions of emotion, leading to some uncertainty regarding whether vocal expressions of emotion will follow similar patterns. However, the heightened amygdala activation for threatening or negative stimuli relative to neutral stimuli^11^ may suggest an increased sensitivity to threatening stimuli will exist, regardless of modality. This may in-turn lead to higher accuracy for negative emotions. In support of this, there is research exploring the reaction of adults with childhood trauma experience to infant cries and reported that childhood neglect specifically was associated with hyperactivation in the anterior and posterior cingulate cortices, insula, and prefrontal and parietal areas^51^ as well as a higher heart rate^52^ compared to individuals without experience of childhood trauma. As cries indicate sadness, which is a negative expression, and individuals with childhood trauma seem to show more sensitivity to the cries than individuals without childhood trauma, it may suggest that the vocal expressions do in fact follow the facial expressions pattern of a bias to negative expressions. Although, whether this patten would be consistent when using similar stimuli (e.g. dynamic facial expressions and linguistic vocal expressions) is unclear.

An important characteristic of real-world emotion recognition includes identifying both subtle and more salient expressions of emotions. Limited evidence exists regarding whether childhood trauma’s effect on emotion recognition accuracy will vary across intensity levels as previous research has explored intensities using morphed stages, which lack ecological validity, rather than genuine intensity levels (e.g. a lower or stronger expression of an emotion). However, as childhood trauma is associated with heightened sensitivities^8^, and less perceptual information needed to recognise negative expressions^5^, it may suggest better accuracy for lower intensity expressions for individuals with more childhood trauma experience compared to individuals without childhood trauma experience. For example, typical populations show poorer accuracy for less intense expressions^53^ but as childhood trauma experience is associated with heightened sensitivities it may lead to expressions being perceived as more intense and in turn lead to better accuracy.

### Interrelated individual differences: alexithymia and psychopathy

Childhood trauma may be linked to certain traits, such as alexithymia and psychopathy traits. In this study, we chose to focus specifically on alexithymia and psychopathy as they have strong links to childhood trauma^12,13^ and emotion recognition performance^14,15^. The effects of both alexithymia and psychopathy on emotion recognition accuracy have been established in both clinical populations^16,17^, as well as in typical populations (e.g. trait level)^18,19^.

Alexithymia has been defined as having no words for feelings and is characterised by difficulties identifying and describing your own feelings, an externally oriented thinking style^20^, and reduced emotional reactivity^21^. Alexithymia traits have been identified as key in the relationship between autism and emotion recognition difficulties^22^. This suggests alexithymia traits can significantly influence emotion deficits associated with related individual differences. Therefore, it may be a possibility that alexithymia traits are a key influence on the relationship between other co-morbid traits (e.g. childhood trauma as this leads to the development of alexithymia traits) and poorer emotion recognition accuracy. Although previous research has supported the strong link between childhood trauma and alexithymia^54^, as well as both their links to emotion deficits^5,19^, they are typically explored separately when exploring emotion performance. This study adds to the current literature by including and controlling for alexithymia traits to ensure childhood trauma experience itself, and not the related alexithymia traits, are responsible for the findings. Previous research exploring alexithymia and facial expressions of emotion reported that higher levels of alexithymia traits were associated with poorer recognition of facial expressions of emotions overall^19^ and needed more perceptual information to recognise static fear expressions^23^; although, for dynamic stimuli, there were no reported differences^23^. For vocal expressions of emotions, Lane et al.^24^ reported that alexithymia traits were associated with poorer recognition of verbal and non-verbal stimuli.

Psychopathy is characterised by four facets: interpersonal (e.g. manipulation), affective (e.g. callousness), lifestyle (e.g. impulsivity), and antisocial traits^25^. It is also associated with emotional and attentional deficits^25^. Similar to the alexithymia literature, research has supported the link between psychopathy and childhood trauma^13^, and their emotion deficits^5,26^, but rarely explores them together. Research exploring psychopathy and childhood trauma in the broader context (e.g. beyond just emotion recognition) reported that 70% of individuals with a high level of psychopathy traits reported severe childhood trauma experience^55,56^. This supports the prevalence of the comorbidity and reasoning for including and controlling for psychopathy when exploring childhood trauma. Previous research exploring psychopathy traits and facial expressions found poorer accuracy for fear, happy, surprise, and sad expressions^26^ as well as for low and high intensity sadness, low intensity disgust and anger, and high intensity fear facial expressions^18^. For vocal expressions of emotion, psychopathy traits were associated with poorer accuracy of fear^26,27^, happy, and surprise vocal expressions^26^.

Experience of childhood trauma may lead to the development of alexithymia traits^12^ and psychopathy traits^13^. Alexithymia may be used as a defence mechanism for childhood trauma^28^. Fang et al.^28^ suggests that by adopting alexithymia traits individuals can alleviate emotional distress by preventing access to internal feelings. For psychopathy, Craparo et al.^13^ suggests that early exposure to trauma may lead to a reduced ability to experience and respond with empathy; a child may become desensitised to future painful/stressful experiences resulting in them becoming less emotionally and physiologically responsive to others^29^. This may lead to the development of psychopathy traits: callousness, lack of empathy, remorse, and guilt^29^. Research has suggested future studies should explore these individual differences together^30^; in research to date they have typically been explored and analysed separately.

Due to the associations and links discussed above, these traits in particular were chosen to include and control for alongside childhood trauma as it is more likely that individuals who have experienced childhood trauma will also present alexithymia and psychopathy traits at a higher level. As higher levels of these traits are also associated with emotion recognition difficulties, isolating which traits may lead to poorer emotion recognition performance is challenging. Therefore, by including and controlling for alexithymia and psychopathy we can investigate the unique contribution childhood trauma has on emotion recognition accuracy.

### The current study

The first aim of this research was to explore the association between childhood trauma and emotion recognition accuracy, and determine if this differed across modality, emotion portrayed, or intensity of stimuli. Emotion recognition studies typically used static emotional faces at one intensity level. We cannot generalise the findings from static images to dynamic stimuli as previous research suggests major differences, and two distinct routes, between them: static images are associated with the right occipital face area, and dynamic stimuli are associated with the right posterior superior temporal sulcus^31^. Also, Starita et al.^23^ found differing emotion recognition performance depending on whether stimuli were static or dynamic; a higher level of alexithymia traits were associated with needing more perceptual information to recognise fearful static faces, but no differences were reported for dynamic stimuli. Static images are also not representative of real-world interactions, as in real conversations we express a mixture of weak and strong intensity dynamic expressions. Furthermore, the experimental paradigm used in this study employed more ecologically valid dynamic stimuli with greater similarity to what would be found in a real-world interaction, three modalities (face, voice, audio-visual), and two intensity levels (normal, strong). A further aim was to examine whether childhood trauma made any independent contributions to emotion recognition accuracy, and if so did this vary across stimulus-based factors.

There are three related research questions: is there a significant effect of childhood trauma on emotion recognition accuracy, when controlling for alexithymia and psychopathy, across (1) different modalities, (2) emotion portrayed, and (3) intensity of stimuli?

It was hypothesised that more experience of childhood trauma would be significantly associated with poorer emotion recognition overall in line with previous research^5,9^. However, due to the strong links between childhood trauma, alexithymia, and psychopathy, it was unclear whether the effects of childhood trauma on emotion recognition would still be significant when controlling for alexithymia and psychopathy.

Regarding the stimulus-based factors, it is hypothesised that childhood trauma would stay consistent across modalities due to the activation of brain areas for negative emotional stimuli^11^. This may suggest that negative emotions will show a similar processing and recognition advantage across all modalities and go beyond modality-specific performance.

For emotion portrayed, it is hypothesised that the effect of childhood trauma on emotion recognition accuracy will vary across emotion portrayed. This hypothesis is informed by the social information processing mechanism^8^ which suggests better accuracy for negative emotions and poorer recognition of positive and neutral emotions (as they are mislabelled as negative ones).

Similar to the modality hypothesis, it is hypothesised that the effect of childhood trauma on emotion recognition accuracy may be consistent across intensity of stimuli due to already heightened sensitivities to negative emotions^8^. This heightened sensitivity may reduce the perceived difference in intensity levels as normal intensity stimuli may be perceived as strong intensity for negative emotions.

Ultimately, the study explores how childhood trauma contributes to emotion recognition accuracy independently of alexithymia and psychopathy, and whether the effect varies across different modalities, emotions, and intensities.

## Materials and methods

### Participants

The final sample consisted of 122 participants (50 female; 71 male; 1 nonbinary, M_age_ = 28 years (18–64), *SD* = 9.42). Twenty-two additional participants were excluded; 11 due to incomplete data, 10 due to their native language not being English, and 1 due to excessively fast reaction times (over 10% of trials had a RT of < 300 ms). Participants were recruited from an online participation site (Testable Minds) in exchange for 9.50 USD, and from an undergraduate psychology course in exchange for 4 course credits. The inclusion criteria were: above 18 years old, normal or corrected-to-normal vision, no significant hearing loss that would render daily tasks and conversations difficult, and English as a first language (as the verbal IQ test uses unusually spelt English words).

### Design

The experimental task variables were modality (face, voice, audio-visual), emotion portrayed (happy, sad, anger, fear, disgust, surprise, neutral), and intensity level (normal, strong). The ‘normal’ intensity expression depicts an average representation of the expression (e.g. a smiling face) and a strong intensity expression depicts a more exaggerated expression (e.g. a wide-open mouth smile). Ultimately, the two intensity levels represent lower and higher intensity expressions. The individual differences variables were childhood trauma, alexithymia and psychopathy.

### Materials

#### Questionnaires

Participants completed three self-report questionnaires to assess: (a) childhood trauma, (b) alexithymia, and (c) psychopathy.

##### Childhood trauma: childhood trauma questionnaire short form (CTQ-SF)

The CTQ-SF^32^ has 28 items each rated from 1 (never true) to 5 (very often true). A higher score on the questionnaire equates to more experience of childhood trauma reported. The subscales are emotional, physical, and sexual abuse, emotional and physical neglect, and minimisation/denial. Participants indicated how often they experienced these situations growing up as a child and teenager, they could skip questions due to the sensitivity of the topic. The CTQ-SF has good internal consistency with high Cronbach Alpha scores across different countries and groups^33^. This was scored according to Bernstein et al.^34^. Reliability of the CTQ-SF in the current sample was analysed, Guttman’s λ_2_ = 0.828.

##### Alexithymia: Toronto Alexithymia questionnaire (TAS-20)

The TAS-20^35^ has 20 items each rated from 1 (strongly disagree) to 5 (strongly agree). A higher score on the questionnaire equates to a higher number of alexithymia traits reported. The subscales are difficulty identifying feelings, difficulty describing feelings, and externally oriented thinking. The TAS-20 has high validity, ease of use, and succinctness^36^. It was scored according to Bagby et al.^35^. Reliability of the TAS-20 in the current sample was analysed, Guttman’s λ_2_ = 0.803.

##### Psychopathy: self report psychopathy scale – short form (SRP-SF)

The SRP-SF^37^ has 29 items each rated from 1 (disagree strongly) to 5 (agree strongly). A higher score on the questionnaire equates to a higher number of psychopathy traits reported. The subscales are: interpersonal, affective, lifestyle, and antisocial items. The SRP-SF was chosen as it demonstrates a ‘satisfactory’ to ‘excellent’ internal consistency and test–retest reliability^38,39^. This was scored according to the Multi-Health Systems Inc.^40^. Reliability of the SRP-SF in the current sample was analysed, Guttman’s λ_2_ = 0.888.

The total scores from each questionnaire were standardised and used in the analyses. The total scores, instead of subscales, were used because of two reasons: (1) the sample size and unevenly distributed numbers in each subscale, and (2) our hypotheses did not specify subscales, we are interested in the overall effect of the individual differences on emotion recognition accuracy.

#### Intelligent quotient verbal task: Wechsler test of adult reading (WTAR)

The WTAR^41^ is a verbal intelligence quotient (IQ) test which includes 50 unusually spelt words. For the online version of the task, words were presented over two pages and participants were audio recorded as they said the words aloud. There was no time limit and scoring followed standard procedure and stopped scoring after 12 wrong pronunciations. The WTAR was chosen because it had been co-normed with the third edition of the Wechsler Adult Intelligence and Memory scales making it the preferred alternative to the National Adult Reading Test^42^. IQ was included to ensure all participants had an IQ score of 80 and above. Participants would have been excluded if their IQ score was categorised as “borderline” or “extremely low”.

#### Emotional tasks: stimuli and procedure

**Figure 1:**
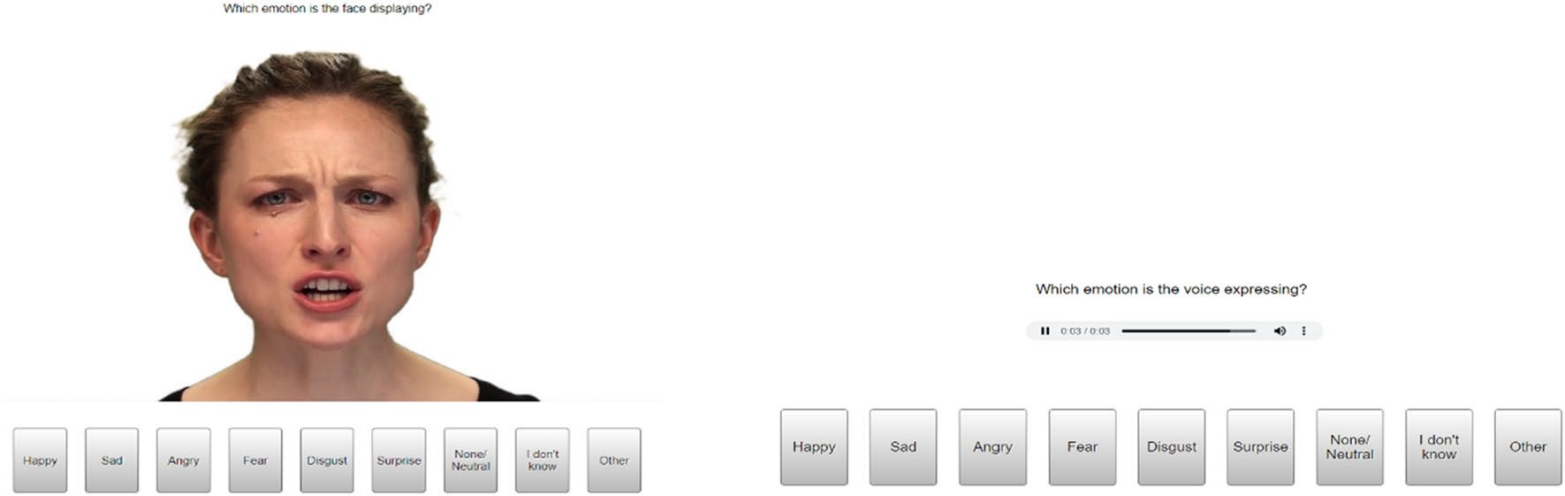
Examples of actor 2 in the face modality (left), the voice modality (right) and the response options.

The stimuli were selected from the Ryerson Audio-Visual Database of Emotional Speech and Song (RAVDESS), a validated database^43^. This database includes audio-visual clips of actors expressing the six basic emotions (happy, sad, angry, fear, surprise, disgust) at two emotional intensity levels of normal and strong, and a neutral condition, across three modalities (faces, voices, and audio-visual). A total of four identities (2 male, 2 female: actors 2, 7, 12, 15) were used in the main task. Three different identities (2 male, 1 female: actors 8, 17, 23) were used for the practice trials. The stimuli in the main task consisted of 13 videos (6 emotions at two intensity levels, plus one neutral) for each modality per actor. The stimuli were videos (the visual and audio-visual conditions) or audio clips (audio condition). The videos show the actor’s faces and the top of their shoulders with black t-shirts on a white background (Fig. 1). For all stimuli the actors recited the sentence “dogs are sitting by the door”, the videos ranged from 3:06 to 4:23 s (*M* = 3.65 s) in duration. In the face condition, participants watched a silent video of one of the actors expressing an emotional or a neutral expression. In the voice condition, no video was presneted, participants listened to a voice displaying an emotional or neutral expression. In the audio-visual condition, participants saw and heard the actors displaying an emotional or neutral expression. The face and voice stimuli were isolated from the audio-visual clip. For example, the happy facial expression would be the audio-visual video but display the visual only, and the happy vocal expression would be the audio-visual video but display the audio only. Therefore, there is no potential difference in expression between the clips. There were a total of 156 trials (13 videos × 4 actors × 3 modalities; 52 trials per modality). Three practice trials preceded the main testing block (with actors not used in the main experiment). The order of the main task and practice trials was randomised.

During the emotion tasks, each stimulus was presented in the middle of the screen along with the response options (Happy, Sad, Fear, Anger, Disgust, Surprise, None/Neutral, I don’t know, and Other) displayed underneath (depicted in Fig. 1). Instructions were displayed above the stimuli and differed per modality: “Which emotion is the face displaying?”, “Which emotion is the voice expressing?”, and “Which emotion are they displaying?”. Participants selected their response by clicking on the appropriate response option. If participants chose a non-emotional answer (i.e. None/Neutral or I don’t know) then the next trial was initiated. If ‘Other’ was chosen they were given the option to free label with the instructions: “Please type what other emotion you think is being expressed in the box”. If one of the basic six emotions was chosen (or after free labelling following an “Other” response), the next screen repeated the video and asked participants to provide an intensity rating. Participants provided their intensity ratings on a 1–10 continuous Likert scale (1: low intensity and 10: high intensity), and responses were provided by clicking on the bar above the number or dragging the slider to the number. On the initial response and intensity rating screens, the stimuli repeated in a loop until the participant responded, there was no time limit.

### General procedure

The experiment was completed online via Qualtrics (for demographics) and Testable (main task, questionnaires, and IQ test). There were four blocks: block 1 was the personality questionnaire, block 2 was the TAS-20 and the face task, block 3 was the SRP-SF questionnaire and the voice task, and block 4 was the CTQ-SF questionnaire and the audio-visual condition. Blocks 2 and 3 were counterbalanced but block 4 was always presented last to ensure the viewing of audio-visual stimuli did not affect recognition in the unimodal conditions. All the emotion tasks had the same procedure, response options, and screens. Breaks were offered after each emotion task, with no set time limit. The final slide redirected participants to an audio calibration to check their microphone was working prior to the WTAR task.

When calculating accuracy scores in the emotion recognition task, free labelled responses and responses of “I don’t know” were classified as incorrect to ensure consistency with how the database labelled the emotions. As four identities were included in the study, generalised mixed models were used to analyse the data to account for any item effects. As a result of this, the stimulus-based variables had to have a reference group. The reference category was audio-visual for modality, neutral for emotion portrayed, and normal intensity for intensity.

### Data analysis

During data pre-processing an analysis of RTs was performed for exclusions. RTs were not included in the main analyses as the dynamic emotion expressions varied in duration and onset. Certain expressions finished quickly (e.g. fear) and others took longer to complete the expression (e.g. sadness). This variation makes it difficult to accurately analyse reaction times. Also, our hypotheses were focused on emotion recognition accuracy (ERA) rather than reaction times. Generalised mixed models were performed (using jamovi, Version 2.3) to examine the role of childhood trauma alone, and whilst controlling for alexithymia and psychopathy, in ERA and if the effect varied across modality, emotion portrayed, or intensity of stimuli. For all models, the random effects were participant and actor.

The dataset generated and analysed during the current study and a fully programmed version of the experiment is available on Open Science Framework: (https://osf.io/vhwxj/?view_only=f6776204f42d4368b4a7a262d0fc8139). 

### Ethics approval

Ethical approval was granted by the Research Ethics Committee for the College of Health, Medicine, and Life Sciences at Brunel University London. Informed consent was obtained from all participants and the research was performed in accordance with relevant guidelines and regulations.

## Results

The descriptives for the experimental task variables and the individual differences variables are presented in Table 1.

**Table 1:** Descriptives table for childhood trauma, alexithymia, and psychopathy displaying the range, mean score, standard deviation, skewness, and kurtosis of the raw total questionnaire scores. Descriptives for modality (faces, voices, audio-visual), emotion portrayed (happy, sad, anger, fear, disgust, surprise, neutral), and intensity (normal, strong) displaying the range, mean and standard deviation of ERA (proprotion correct).

Variables	Range	Mean score	Standard deviation	Skewness	Kurtosis
Childhood trauma	75 (25–100)	42.30	14.15	1.27	1.92
Alexithymia	60 (25–85)	49.81	12.25	0.06	− 0.56
Psychopathy	74 (29–103)	55.90	14.65	0.58	0.25
Emotion tasks (response accuracy)					
Modality					
Faces	0.54 (0.40–0.94)	0.73	0.44		
Voices	0.52 (0.31–0.83)	0.60	0.49		
Audio-visual	0.60 (0.38–0.98)	0.84	0.37		
Emotion					
Happy	0.67 (0.33–1.00)	0.80	0.40		
Sad	0.83 (0.17–1.00)	0.70	0.46		
Anger	0.79 (0.21–1.00)	0.83	0.38		
Fear	0.87 (0.13–1.00)	0.72	0.45		
Disgust	0.71 (0.25–0.96)	0.65	0.48		
Surprise	0.71 (0.21–0.92)	0.63	0.48		
Neutral	0.83 (0.17–1.00)	0.77	0.42		
Intensity					
Normal	0.46 (0.43–0.89)	0.70	0.46		
Strong	0.52 (0.42–0.94)	0.75	0.43		

### Is childhood trauma alone associated with emotion recognition accuracy?

For all models, the random effects were participant and actor. Bonferroni correction has been applied.

#### Does this vary across modality?

**Figure 2:**
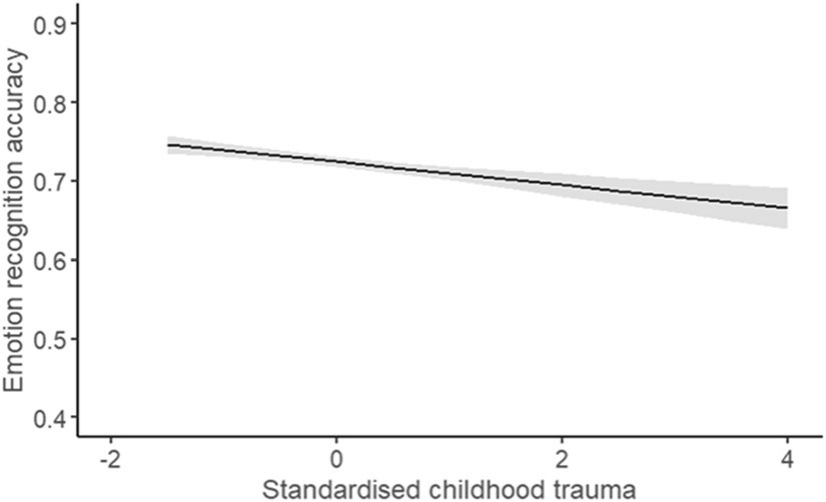
The average ERA (proportion correct) for the standardised total score of childhood trauma (derived from the CTQ-SF). The shaded area represents the 95% confidence interval.

A generalised mixed model was employed to explore the effect of childhood trauma on ERA. The fixed factors were childhood trauma and modality. The fixed factors had a significant main effect of childhood trauma, X^2^ (1) = 4.33, *p* = 0.001, β =  − 0.09, exp(B) = 0.92, and modality, X^2^ (2) = 121.67, *p* < 0.001, with β =  − 0.71 and exp(B) = 0.49 for faces and β =  − 1.35 and exp(B) = 0.26 for voices compared to audio-visual. These are small effect sizes according to Chen et al.^44^, who suggested an odds ratio (in this case exp(B)) below 1.68 was small. The higher score of childhood trauma, indicating more childhood trauma experience, was associated with poorer ERA, z = − 2.08, *p* = 0.001 (Fig. 2). Accuracy was significantly better for audio-visual emotions compared to facial emotions, z = − 9.49, *p* < 0.001, and vocal emotions, z = 5.98, *p* < 0.001. There was not a significant interaction between childhood trauma and modality, X^2^ (2) = 2.79, *p* = 0.248, with β = 0.08 and exp(B) = 1.09 for childhood trauma * faces – audio-visual and β = 0.06 and exp(B) = 1.06 for childhood trauma * voices – audio-visual. This suggests the effect of childhood trauma did not vary significantly across modalities.

#### Does this vary across emotion portrayed?

A generalised mixed model was employed to explore the effect of childhood trauma on ERA. The fixed factors were childhood trauma and emotion portrayed. The fixed factors had a significant main effect of childhood trauma, X^2^ (1) = 5.71, *p* = 0.001, β =  − 0.10, exp(B) = 0.90 and emotion portrayed, X^2^ (6) = 83.87, *p* < 0.001 (a complete table with β and exp(B) for each emotion portrayed is presented in Appendix A). More experience of childhood trauma was associated with poorer ERA overall, z = − 2.39, *p* = 0.001. There was a significant difference in accuracy for fear expressions compared to neutral expressions, with fear expressions having significantly poorer accuracy, z = − 2.73, *p* = 0.002. There was not a significant interaction between childhood trauma and emotion portrayed, X^2^ (6) = 3.99, *p* = 0.226 (β and exp(B) for each interaction is presented in Appendix A). This suggests the effect of childhood trauma did not vary significantly across emotions.

#### Does this vary across intensity?

A generalised mixed model was employed to explore the effect of childhood trauma on ERA. The fixed factors were childhood trauma and intensity of stimuli. The fixed factors had a significant main effect of childhood trauma, X^2^ (1) = 4.02, *p* = 0.015. β =  − 0.08, exp(B) = 0.92. There was not a significant main effect of intensity, X^2^ (1) = 2.35, *p* = 0.060, β = 0.34, exp(B) = 1.40. More experience of childhood trauma experience was associated with poorer ERA, z = − 2.00, *p* = 0.015. However, ERA was comparable whether the stimuli were normal or strong intensity, z = 1.53, *p* = 0.060. There was not a significant interaction between childhood trauma and intensity, X^2^ (1) = 0.00, *p* = 0.322, β =  − 0.00, exp(B) = 1.00. This suggests the effect of childhood trauma did not vary significantly depending on whether the stimuli were normal or strong intensity.

### Is childhood trauma associated with emotion recognition accuracy when controlling for alexithymia and psychopathy?

For all models, the random effects were participant and actor. Bonferroni correction has been applied.

#### Does this vary across modality?

**Figure 3:**
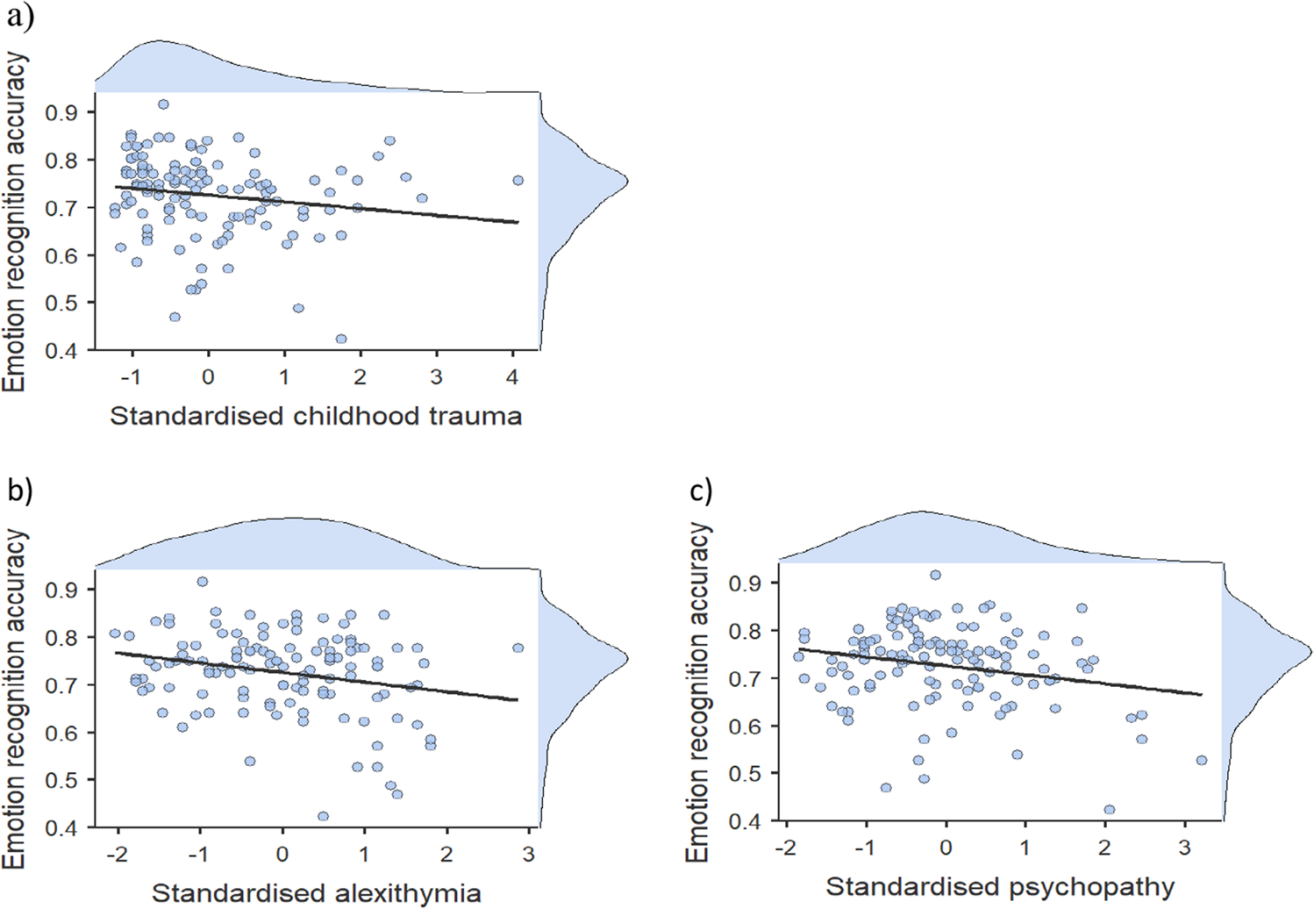
Plots showing the average ERA for the standardised total scores of (**a**) childhood trauma (derived from the CTQ-SF), (**b**) alexithymia (derived from the TAS-20), and (**c**) psychopathy (derived from the SRP-SF): higher scores indicating more experience of childhood and a higher level of traits of alexithymia and psychopathy.

**Figure 4:**
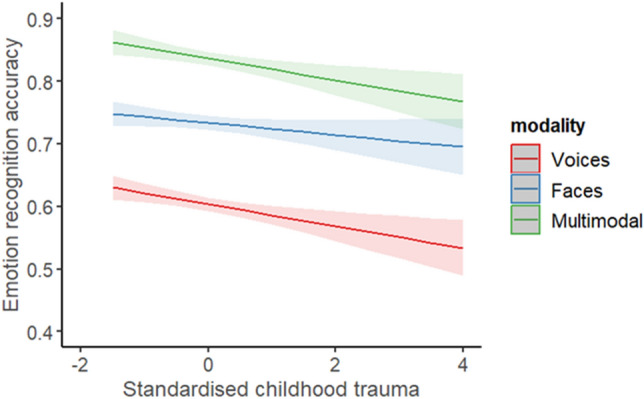
Average ERA of the standardised total score for childhood trauma (derived from the CTQ-SF) across modalities. The shaded area represents the 95% confidence interval.

A generalised mixed model was employed to explore the effect of childhood trauma on ERA. The fixed factors were childhood trauma and modality and the covariates were alexithymia and psychopathy. After controlling for alexithymia and psychopathy, childhood trauma was no longer significant, X^2^ (1) = 2.96, *p* = 0.085 (Fig. 3). Also not significant were alexithymia, X^2^ (1) = 3.81, *p* = 0.051, and psychopathy, X^2^ (1) = 2.10, *p* = 0.148 (Fig. 3). However, modality was still significant, X^2^ (2) = 121.32, *p* < 0.001, with significantly better accuracy for audio-visual emotions compared to facial and vocal emotions. There was not a significant interaction between childhood trauma and modality. This suggests the effect of childhood trauma did not vary significantly across the three modalities (Fig. 4). Fixed effects parameter estimates are presented in Table 2. When comparing the AIC and BIC values for the original modality analysis (childhood trauma alone) with the current analysis (controlling for alexithymia and psychopathy), the AIC was lower for the current model (20,805.11) compared to the original analysis (20,808.71), but the BIC was higher for the current model (20,962.19) compared to the original analysis (20,950.08). As well as this, model comparisons between the original model and the current model showed a significantly better fit to the data for the current model, X2 (2) = 8.52, p = 0.001.

**Table 2:** The fixed effects parameter estimates table for ERA for childhood trauma, modality, alexithymia, and psychopathy.

Fixed effects	Estimate	Standard error	exp(B)	95% CI (lower, upper)		z	*p*
Intercept	1.10	0.15	3.01	2.26	4.00	7.57	< 0.001*
Childhood trauma	− 0.07	0.04	0.93	0.86	1.01	− 1.72	0.085
Faces (faces–audio-visual)	− 0.71	0.07	0.49	0.42	0.57	− 9.48	< 0.001*
Voices (voices–audio-visual)	− 1.35	0.23	0.26	0.17	0.40	− 5.98	< 0.001*
Alexithymia	− 0.07	0.04	0.93	0.86	1.00	− 1.95	0.051
Psychopathy	− 0.06	0.04	0.95	0.88	1.02	− 1.45	0.148
Childhood trauma * faces	0.08	0.05	1.09	0.99	1.20	1.67	0.096
Childhood trauma * voices	0.06	0.05	1.06	0.96	1.17	1.15	0.248

*Represents significant values (*p* < 0.05).

#### Does this vary across emotion portrayed?

**Figure 5:**
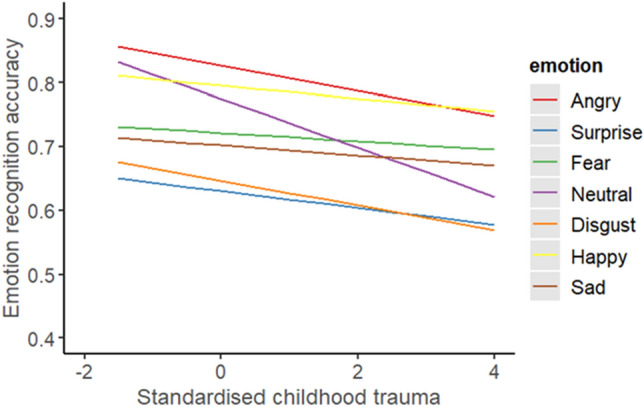
Average ERA of a standardised total score for childhood trauma (derived from the CTQ-SF) across emotion portrayed.

A generalised mixed model was employed to explore the effect of childhood trauma on ERA. The fixed factors were childhood trauma and emotion portrayed and the covariates were alexithymia and psychopathy. After controlling for alexithymia and psychopathy, childhood trauma was still significant, X^2^ (1) = 4.27, *p* = 0.001. There was also a significant main effect of emotion portrayed, X^2^ (6) = 85.12, *p* < 0.001. There was not a significant effect of alexithymia, X^2^ (1) = 1.44, *p* = 0.115, or psychopathy, X^2^ (1) = 1.33, *p* = 0.125. There was not a significant interaction between childhood trauma and emotion portrayed, X^2^ (6) = 3.99, *p* = 0.339. This suggests the effect of childhood trauma did not vary significantly across which emotion was expressed (Fig. 5). Fixed effects parameter estimates are presented in Table 3. A full table of fixed effects parameter estimates, including all interactions, is presented in Appendix B. The AIC and BIC values for the original analysis exploring emotion portrayed (childhood trauma alone) were lower (AIC = 19,743.71; BIC = 20,293.48) than the current model (controlling for alexithymia and psychopathy) (AIC = 19,744.17; BIC = 20,309.65). Model comparisons between the original model and the current model showed a significantly better fit to the data for the current model, X2 (2) = 9.10, p = 0.001.

**Table 3:** The fixed effects parameter estimates table for ERA for childhood trauma, emotion portrayed, alexithymia, and psychopathy.

Fixed effects	Estimate	Standard error	exp(B)	95% CI (lower, upper)		z	*p*
Intercept	1.21	0.12	3.37	2.66	4.26	10.09	< 0.001*
Childhood trauma	− 0.09	0.04	0.91	0.84	1.00	− 2.07	0.001*
Happy (happy–neutral)	− 0.13	0.17	0.88	0.63	1.24	− 0.73	0.233
Sad (sad–neutral)	− 0.55	0.39	0.58	0.27	1.25	− 1.39	0.008
Angry (angry–neutral)	0.40	0.64	1.50	0.43	5.27	0.63	0.264
Fear (fear–neutral)	− 0.52	0.19	0.60	0.41	0.86	− 2.76	0.003*
Disgust (disgust–neutral)	− 0.89	0.61	0.41	0.13	1.35	− 1.46	0.072
Surprise (surprise–neutral)	− 0.93	0.66	0.39	0.11	1.43	− 1.41	0.079
Alexithymia	− 0.06	0.05	0.95	0.86	1.04	− 1.20	0.115
Psychopathy	− 0.06	0.05	0.95	0.86	1.04	− 1.15	0.125

*Represents significant values (*p* < 0.05).

#### Does this vary across intensity?

**Figure 6:**
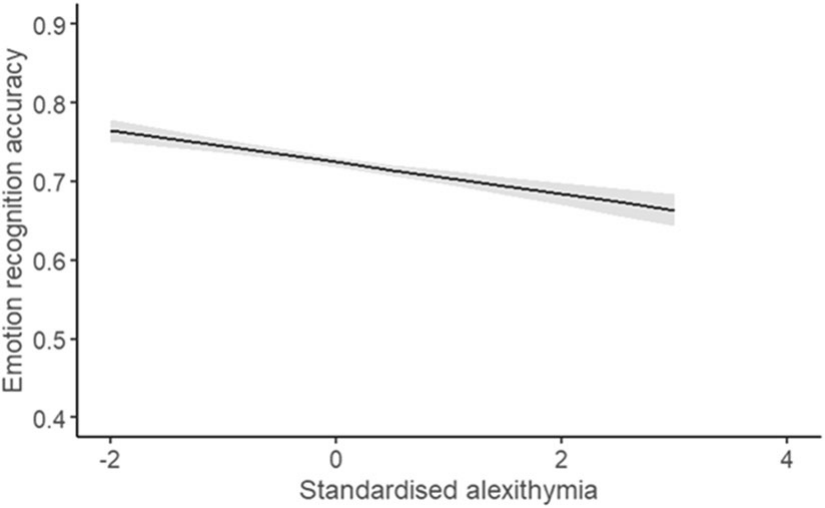
The average ERA for the standardised total score of alexithymia (derived from the TAS-20). The shaded area represents the 95% confidence interval.

**Figure 7:**
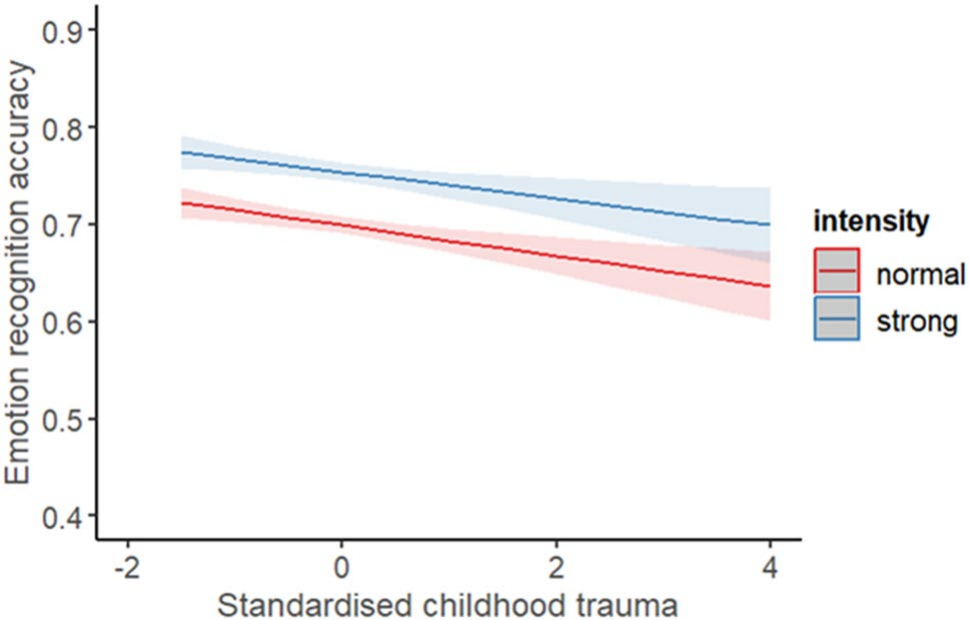
Average ERA of a standardised total score for childhood trauma (derived from the CTQ-SF) across intensity. The shaded area represents the 95% confidence interval.

A general mixed model was employed to explore the effect of childhood trauma on ERA. The fixed factors were childhood trauma and intensity and the covariates were alexithymia and psychopathy. After controlling for alexithymia and psychopathy, childhood trauma was no longer significant, X^2^ (1) = 2.56, *p* = 0.055. Intensity was not significant, X^2^ (1) = 2.35, *p* = 0.063, and neither was psychopathy, X^2^ (1) = 2.06, *p* = 0.076. However, alexithymia was significant, X^2^ (1) = 4.19, *p* = 0.021 (Fig. 6). There was not a significant interaction between childhood trauma and intensity, X^2^ (1) = 0.00, *p* = 0.484. This suggests the effect of childhood trauma did not vary significantly depending on whether the stimuli were normal or strong intensity (Fig. 7). Fixed effects parameter estimates are presented in Table 4. The AIC for the current analysis (childhood trauma alone) was lower (21,655.47) than the original model exploring intensity (controlling for alexithymia and psychopathy) (21,659.53), but the BIC was higher for the current model (21,749.72) compared to the original analysis (21,738.07). Model comparisons between the original model and the current model showed a significantly better fit to the data for the current model, X2 (2) = 9.10, p = 0.001.

**Table 4:** The fixed effects parameter estimates table for ERA for childhood trauma, intensity, alexithymia, and psychopathy.

Fixed effects	Estimate	Standard error	exp(B)	95% CI (lower, upper)		z	*p*
Intercept	1.04	0.14	2.84	2.17	3.71	7.58	< 0.001*
Childhood trauma	− 0.06	0.04	0.94	0.87	1.01	− 1.60	0.055
Intensity (strong–normal)	0.34	0.22	1.40	0.91	2.16	1.53	0.063
Alexithymia	− 0.08	0.04	0.92	0.86	1.00	− 2.05	0.021*
Psychopathy	− 0.06	0.04	0.95	0.88	1.02	− 1.44	0.076
Childhood trauma * intensity	− 0.00	0.03	1.00	0.93	1.07	− 0.04	0.484

*Represents significant values (*p* < 0.05).

## Discussion

The main aim of this study was to investigate whether childhood trauma, when controlling for interrelated individual differences, was associated with ERA. Furthermore, we were interested in how this effect might vary across modality, emotion portrayed, and intensity.

We examined the effects of childhood trauma alone on ERA in three analyses: one exploring the effects of modality; one examining the effects of emotion portrayed; another exploring the effects of intensity. The analyses revealed that childhood trauma alone was associated with poorer ERA, but this did not differ significantly across modality, emotion portrayed, or intensity. We also examined the effects of childhood trauma on accuracy whilst controlling for alexithymia and psychopathy in three analyses: exploring the effects of modality, emotion portrayed, and intensity. The analyses revealed that childhood trauma was associated with poorer accuracy when exploring emotion portrayed, but this did not significantly differ across emotions. The analyses exploring the effects of modality and intensity revealed childhood trauma was no longer significantly associated with poorer accuracy.

### Childhood trauma alone

The ERA findings when examining childhood trauma alone generally align with previous findings. There was a significant association between childhood trauma and ERA across all three analyses. In line with previous research^5,9^, individuals who reported more experience of childhood trauma showed significantly poorer ERA compared to those reporting less experience of childhood trauma. There were no significant interactions, suggesting the effect of childhood trauma was consistent across stimulus-based factors. This suggests the emotion recognition deficit associated with childhood trauma is not exacerbated or ameliorated when viewing dynamic stimuli varying in modality, emotion portrayed, or intensity. The expected findings of childhood trauma being associated with emotion recognition performance differed when controlling for interrelated individual differences.

### Childhood trauma when controlling for alexithymia and psychopathy

In two of our analyses – those examining the relationship between childhood trauma and (1) modality, and (2) intensity – including covariates in the model resulted in the relationship between childhood trauma and ERA no longer being significant. Consequently, it is likely that the results of the original analyses (childhood trauma alone) may have been significantly influenced by the interrelated individual differences. As childhood trauma was no longer significant itself when controlling for the other traits, it may suggest that they were significantly contributing to the original model. This shows the importance of future research controlling for co-morbid traits when exploring individual differences.

The current study is one of the few to explore childhood trauma’s effect across different modalities. There was not a significant interaction between modality and childhood trauma. There was also no longer a significant effect of childhood trauma when exploring intensity. Although, alexithymia was significant. Individuals who reported higher levels of alexithymia traits were associated with poorer ERA, following previous findings^19,23^. This may suggest that the original analyses (childhood trauma alone) included and presented the significant contribution of alexithymia alongside childhood trauma. This raises the possibility that present theories linking childhood trauma and emotion recognition deficits may need to consider other related factors. However, it is important to note that accuracy is only one way to assess emotion processing: future research may benefit from exploring childhood trauma and other, more subtle measures of emotion recognition, such as perceived intensity ratings across intensities.

After controlling for alexithymia and psychopathy, childhood trauma was still significant when exploring emotion portrayed. This suggests that childhood trauma itself was significantly associated with accuracy when exploring emotion portrayed and not interrelated individual differences. There was not a significant interaction between childhood trauma and emotion portrayed, differing from our hypothesis and previous research^5,9^. This difference may be because we used dynamic stimuli which are easier to recognise as they closer represent real world emotions and provide more emotion cues than a static face^45^. Therefore, it may have attenuated some of the difficulties associated with specific emotions. Although, our lack of interaction is somewhat supported by a meta-analysis^10^ which reported poorer accuracy for fearful and happy expressions only if childhood trauma was recent (less than 2 years ago). As we tested an adult sample, it is unlikely that the experience of childhood trauma would be recent. The current findings suggest that experience of childhood trauma is associated with poorer accuracy across all emotions, not just positive or neutral.

The current findings support the inclusion of strongly associated individual differences as childhood trauma was no longer significant when exploring modality and intensity. This suggests the original analyses (childhood trauma alone) exploring modality and intensity may have also reported the related traits’ contribution to the emotion deficits. For intensity, alexithymia had the largest influence based on the data.

The findings suggest that the relationship between childhood trauma and emotion recognition accuracy, when exploring intensity, may be significantly influenced by other related factors – in this case alexithymia. This extends our understanding of the relationship between childhood trauma and emotion deficits as we can start investigating these in a similar way to the emotion deficits associated with alexithymia. This may provide clues on how to remediate the associated emotion recognition deficits.

### Limitations and future directions

Even though the study sought to address stimuli limitations by using dynamic stimuli, some stimulus-based limitations remained. For one, all the actors were Caucasian. From our sample, 30% of participants identified themselves as Caucasian with the majority (51%) identifying as Asian/Pacific Islander. Previous research regarding whether there is an own-ethnicity bias (viewing actors who match your ethnicity improves your performance) is inconsistent. Some papers have reported there is an own-ethnicity bias^46,47^ in which case the Caucasian participants would have an advantage. However, other research has found there is no own-ethnicity bias^48,49^ which would reduce the issue of using all Caucasian stimuli. Another limitation could be not exploring childhood trauma subscales or other participant variables such as sex or age. However, we analysed and controlled for the relevant variables in line with our hypotheses. Having established that alexithymia and psychopathy traits may underpin some of the emotion recognition differences that are observed following experience of childhood trauma, future research may explore whether these relationships are driven by certain subscales in the alexithymia and psychopathy questionnaires.

Finally, as mentioned above, future research may benefit from exploring more sensitive measures of emotion recognition (e.g. perceived intensity ratings and a measure of sensitivity to emotional intensity—due to childhood trauma’s association with heightened sensitivities). Future research exploring individual differences should control for other interrelated ones to ensure the main factor is the effect being explored.

## Conclusion

To conclude, this study extended previous research on the association between childhood trauma and emotion recognition; we included multiple dynamic modalities and varying intensities of emotional stimuli, and controlled for other related individual differences (alexithymia and psychopathy traits). Childhood trauma alone had a significant association with ERA when exploring modality, emotion portrayed, and intensity. More experience of childhood trauma was associated with poorer accuracy. Notably, when controlling for alexithymia and psychopathy, childhood trauma only had a significant association with poorer accuracy when exploring emotion portrayed. This illustrates the importance of including and controlling for interrelated individual differences. It may suggest that present theories involving childhood trauma and emotion deficits may need to account for factors such as higher levels of alexithymia and psychopathy traits in the groups being studied. It could also suggest that we may be able to explore the emotion deficits associated with childhood trauma and alexithymia similarly and this may in turn provide clues to alleviate the associated deficits.

### Supplementary Information

Supplementary Information.

## Data Availability

The dataset generated and analysed during the current study and a fully programmed version of the experiment is available on Open Science Framework: (https://osf.io/vhwxj/?view_only=f6776204f42d4368b4a7a262d0fc8139).
