# Supplementary material for: The association between childhood trauma and emotion recognition is reduced or eliminated when controlling for alexithymia and psychopathy traits
Source: Sci Rep. 2024 Feb 10;14:3413. doi: 10.1038/s41598-024-53421-5 (PMC10858958; doi:10.1038/s41598-024-53421-5)
Supplement: Supplementary file 1 — Supplementary Information. [file 41598_2024_53421_MOESM1_ESM.pdf]

Appendices

Appendix A

Complete table of fixed effects parameter estimates for childhood trauma and emotion portrayed

Fixed effects	Estimate	Standard Error	exp(B)	95% CI (lower, upper)		*z*	*p*
Intercept	1.21	0.12	3.37	2.65	4.27	10.02	<.001
Childhood trauma	-0.10	0.04	0.90	0.83	0.98	-2.39	.017
Happy (Happy – Neutral)	-0.13	0.17	0.87	0.62	1.23	-0.77	.441
Sad (Sad – Neutral)	-0.54	0.39	0.58	0.27	1.26	-1.38	.168
Angry (Angry – Neutral)	0.41	0.65	1.51	0.43	5.35	0.64	.524
Fear (Fear – Neutral)	-0.52	0.19	0.60	0.41	0.86	-2.73	.006
Disgust (Disgust – Neutral)	-0.89	0.61	0.41	0.13	1.35	-1.46	.143
Surprise (Surprise – Neutral)	-0.93	0.66	0.39	0.11	1.44	-1.41	.158
Childhood trauma * Happy	0.18	0.14	1.20	0.91	1.58	1.29	.196
Childhood trauma * Sad	0.19	0.15	1.21	0.90	1.63	1.26	.206
Childhood trauma * Angry	0.09	0.15	1.10	0.81	1.48	0.60	.546
Childhood trauma * Fear	0.22	0.14	1.24	0.94	1.64	1.53	.125
Childhood trauma * Disgust	0.14	0.13	1.16	0.89	1.50	1.08	.282
Childhood trauma * Surprise	0.17	0.14	1.19	0.91	1.55	1.24	.215

Appendix B

Complete table of fixed effects parameter estimates for childhood trauma, emotion portrayed, alexithymia, and psychopathy

Fixed effects	Estimate	Standard Error	exp(B)	95% CI (lower, upper)		z	*p*
Intercept	1.21	0.12	3.37	2.66	4.26	10.09	<.001
Childhood trauma	-0.09	0.04	0.91	0.84	1.00	-2.07	.039
Happy (Happy – Neutral)	-0.13	0.17	0.88	0.63	1.24	-0.73	.465
Sad (Sad – Neutral)	-0.55	0.39	0.58	0.27	1.25	-1.39	.164
Angry (Angry – Neutral)	0.40	0.64	1.50	0.43	5.27	0.63	.528
Fear (Fear – Neutral)	-0.52	0.19	0.60	0.41	0.86	-2.76	.006
Disgust (Disgust – Neutral)	-0.89	0.61	0.41	0.13	1.35	-1.46	.143
Surprise (Surprise – Neutral)	-0.93	0.66	0.39	0.11	1.43	-1.41	.157
Alexithymia	-0.06	0.05	0.95	0.86	1.04	-1.20	.230
Psychopathy	-0.06	0.05	0.95	0.86	1.04	-1.15	.249
Childhood trauma * Happy	0.18	0.14	1.20	0.91	1.59	1.29	.197
Childhood trauma * Sad	0.19	0.15	1.21	0.90	1.63	1.26	.206
Childhood trauma * Angry	0.09	0.15	1.10	0.81	1.48	0.61	.545
Childhood trauma * Fear	0.22	0.14	1.24	0.94	1.64	1.53	.125
Childhood trauma * Disgust	0.14	0.13	1.15	0.89	1.50	1.07	.283
Childhood trauma * Surprise	0.17	0.14	1.19	0.91	1.55	1.24	.215
